# Prevalence, Risk Factors, and Negative Outcomes of Anxiety and Depressive Disorders among HIV-Infected Children and Adolescents in Uganda: CHAKA Study 2014-2017

**DOI:** 10.1155/2022/8975704

**Published:** 2022-05-05

**Authors:** Richard Stephen Mpango, Wilber Ssembajjwe, Godfrey Zari Rukundo, Tatiana Taylor Salisbury, Jonathan Levin, Kenneth D. Gadow, Vikram Patel, Eugene Kinyanda

**Affiliations:** ^1^Mental Health Project, MRC/UVRI and LSHTM Uganda Research Unit, Entebbe, Uganda; ^2^Brown School, Washington University in St. Louis, Missouri 63130, USA; ^3^Department of Mental Health, School of Health Sciences, Soroti University, Soroti, Uganda; ^4^Statistical Section, MRC/UVRI and LSHTM Uganda Research Unit, Entebbe, Uganda; ^5^Department of Psychiatry, Mbarara University of Science and Technology, Uganda; ^6^Health Service and Population Research Department, Institute of Psychiatry, Psychology and Neuroscience, King's College London, UK; ^7^School of Public Health, Faculty of Health Sciences, University of Witwatersrand, Johannesburg, South Africa; ^8^Department of Psychiatry, Stony Brook University, Stony Brook, York, USA; ^9^Department of Global Health and Social Medicine, Harvard Medical School, Massachusetts, USA; ^10^Department of Psychiatry, Makerere College of Health Sciences, Kampala, Uganda

## Abstract

**Background:**

Children and adolescents infected with HIV/AIDS (CA-HIV) experience a considerable burden of depressive and anxiety disorders that have a tendency to persist into adulthood. The aim of this study was to determine the prevalence of anxiety, depression, and their clinical correlates among children and adolescents with HIV/AIDS (CA-HIV) in Uganda.

**Methods:**

A random sample of 1339 CA-HIV (ages 5-18 years) and their caregivers completed a standardized *DSM-*5*-*referenced psychiatric rating scale, the Child and Adolescent Symptom Inventory-5 (CASI-5). The prevalence of “anxiety and depression” was estimated at 95% confidence intervals. Logistic and ordinal regression models were fitted for the clinical correlates and clinical outcomes.

**Results:**

The overall prevalence of “any anxiety and depressive disorders” was 13.7% at 95% CI (based upon the symptom count criteria); 4.0% (95% CI) met the clinical psychiatric disorder criteria (both symptom count and functional impairment criteria). Anxiety disorder was more prevalent (9%, 95% CI) than depression (6.4%, 95% CI). Correlates of “anxiety and depressive disorders” included age of the child, caregiver' psychological distress, caregivers' age, child-caregiver relationship, and child's current CD4 count (aOR1.00, 95% CI 1.02–1.05; *p* = 0.021). Anxiety disorders (aOR 2.58, 95% CI 1.16-5.42; *p* = 0.02) and depressive disorders (aOR 2.47, 95% CI 1.93–6.52; *p* = 0.041) were also associated with hospital admissions. *Limitations.* Analyses were cross-sectional; we cannot comment on the causal directions. The results are entirely based upon caregiver' reports.

**Conclusions:**

There is an urgent need to integrate mental health services into routine HIV care for CA-HIV in sub-Saharan Africa.

## 1. Introduction

Children and adolescents infected with HIV/AIDS (CA-HIV) experience a considerable burden of depressive and anxiety disorders [1–3]. If left untreated, these disorders have a tendency to persist into adulthood, resulting into adverse effects on physical and mental health, life adjustment, and functional outcomes [1–11]. A systematic review of eight studies published before 2006 in high income countries among CA-HIV reported an average prevalence of 24.3% and 25% for anxiety and depressive disorder, respectively [4]. More recent studies in high income countries have reported prevalence of emotional problems of 25% to 38% [1, 5, 6]. In Africa, studies conducted among CA-HIV have reported rates of emotional problems ranging from 9% to 63% [3, 9–11]. Although sub-Saharan Africa carries the biggest burden of HIV/AIDS, there is little information concerning the burden of anxiety and depression, as well as the associated disabilities and negative consequences among children and adolescents. The aim of this study was to determine the prevalence of anxiety, depression, and their clinical correlates among children and adolescents with HIV/AIDS (CA-HIV) in Uganda. This study was conducted as part of a larger study (*CHAKA* study) that determined the mental health problems among HIV infected children and adolescents in Kampala and Masaka in Uganda. The interest in anxiety and depressive disorders was due to the associated disabilities and other negative consequences [1, 2, 12–14].

## 2. Methods

### 2.1. Participants and Setting

This study was undertaken at five HIV clinics in rural Masaka district (The AIDS Support Organisation (TASO) clinic, the Kitovu Mobile AIDS Organisation, and the Uganda Cares clinic) and urban Kampala City Council Authority (Joint Clinical Research Centre (JCRC) clinic and Nsambya Homecare Department) in Uganda. A sample of 1,339 child/adolescent-caregiver dyads with each site expected to contribute an equal number of 268 study participants. Study eligibility criteria included the following: (i) CA-HIV aged between 5 and 17 years of age, with children defined as aged between 5 and 11 years and adolescents defined as aged between 12 and17 years; (ii) caregivers older than 17 years of age; (iii) both able to speak English or Luganda (the local language spoken in the study areas); and (iv) both expected to remain in the study area for the subsequent 12 months. Exclusion criteria were as follows: (i) concurrent enrolment in another study (this applied to only one study site, the Joint Clinical Research Centre site); (ii) sick and in need of immediate medical attention; and (iii) those unable to understand the study instruments for whatever reason. Eligible study participants provided informed consent (caregiver) and assent (CA-HIV) after explanation of the study objectives and procedures.

### 2.2. Procedure

The research assistants identified potential participants from the patients' registers. Those who met eligibility criteria were then invited to enroll into the study, and they were recruited consecutively. This procedure was repeated each clinic day until the required sample number was attained. The study protocol was administered by trained psychiatric nurses and psychiatric clinical officers supervised by a clinical psychologist (RM) and a psychiatrist (EK).

### 2.3. Measures

The assessment tools such as the Child and Adolescent Symptom Inventory-5 (CASI-5) [15] used for the first time in Uganda were locally adapted [16]. Study variables reported in this paper are described in Table 1.

The CASI-5 was administered to derive (a) the Symptom Count Cutoff score (yes/no; categorical model) which indicates whether a CA-HIV has the prerequisite number of symptoms necessary for a *DSM-5* diagnosis, (b) Impairment Cutoff score (yes/no; categorical model) which indicates whether the CA-HIV is impaired by the symptoms of a particular disorder (rating of *often* or *very often*) regardless of the number or severity of symptoms, and (c) the Clinical Cutoff score (yes/no; categorical model) which is a combination of the Symptom Count Cutoff score and the Impairment Cutoff score [15]. For the Symptom Count Score method, each item (i.e., symptom) in the CASI-5 is recoded as either present [1] or absent (0), which requires a modification of item weights as follows: never = 0, sometimes = 0, often = 1, very often = 1, and no = 0 and yes = 1. If a CA-HIV exhibited the minimum number of symptoms necessary for a diagnosis of a disorder, the CA-HIV received a Symptom Count Cutoff Score of yes, which indicated that a more in-depth clinical evaluation was warranted. The last item in each symptom category assessed the degree to which the symptoms of a specific disorder interfere with school or social functioning. Responses are scored never = 0, sometimes = 0, often = 1, and very often = 1. If a CA-HIV obtained a score of “1,” then he/she obtained an Impairment Cutoff score (yes) regardless of his/her Symptom Count Cutoff score. If a CA-HIV had both a Symptom Count Cutoff score (yes) and an Impairment Cutoff score (yes) for the same symptom category, then he/she received a Clinical Cutoff score (yes). A Clinical Cutoff score indicates that the CA-HIV has the prerequisite symptoms of the disorder and is impaired [15, 16].

### 2.4. Statistical Analysis

**Figure 1:**
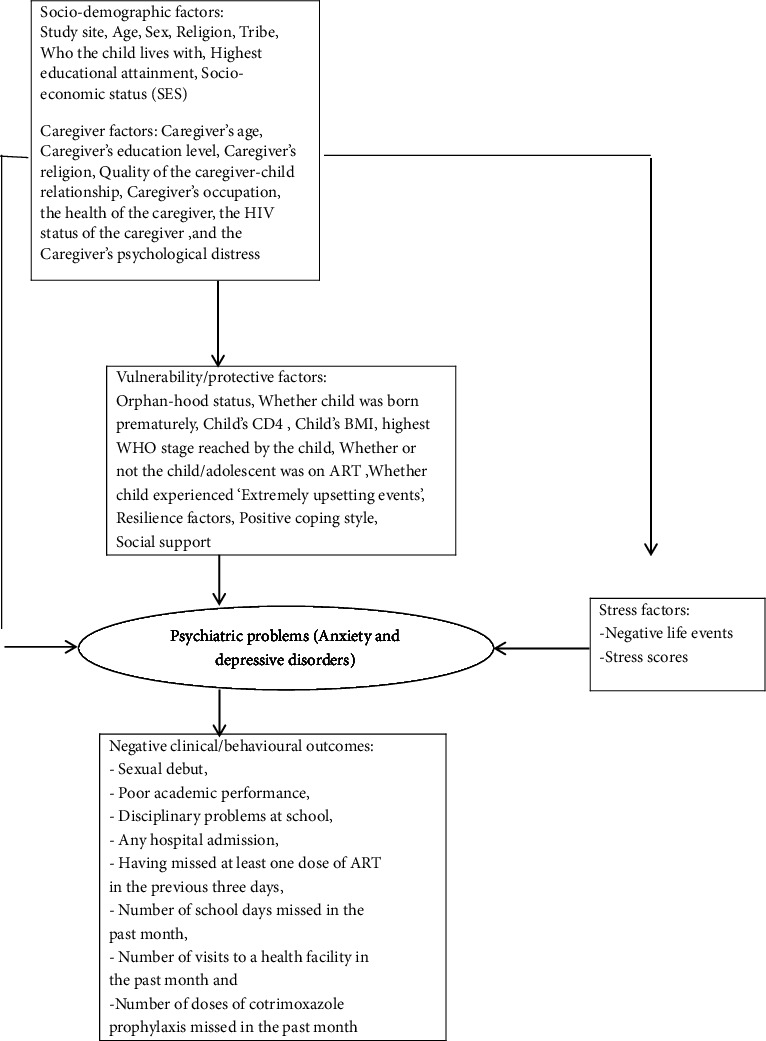
Conceptual framework based on the stress vulnerability models for anxiety and depressive disorders [18].

Factors associated with each of the two disorders (anxiety and depressive disorders) were found by fitting separate logistic regression models. We used the model building approach recommended by Victora et al. [17], with a conceptual framework based upon the stress vulnerability model (see Figure 1) [18].

Initially, a prediction model was selected based only on the sociodemographic factors. All models included study site (as a design variable), sex, and age as *á priori* confounders. Potential additional sociodemographic variables were education level, socioeconomic status (found using principal component analysis based on a prespecified set of consumer durables as proposed by Vyas and Kumaranayake) [19], tribe of the child (Muganda vs. non-Muganda), and religion of the child (Christian, Muslim, or “other”). The additional sociodemographic variables were removed from the model if they were not statistically significant at the 15% level, using a stepwise (backward elimination) algorithm as recommended by Vittinghoff et al. [20]; the use of a liberal *p* value of 0.15 is to ensure that all confounders are included in the model.

At the second stage, the following caregiver factors were considered for inclusion: caregiver's age, caregiver's education level, caregiver's religion, caregiver's employment, the health of the caregiver, the HIV status of the caregiver, and the caregiver's score on the Self-Report Questionnaire-20 (SRQ-20) scale [21] (with higher scores denoting more psychological distress). All caregiver variables were added to the chosen sociodemographic model, and those that were not statistically significant at the 15% level were removed using a backward elimination algorithm.

At the third stage of model building, variables reflecting the psychosocial environment (or vulnerability/protective variables) were considered for inclusion. The candidate variables were orphan-hood status (no parent alive, one parent alive, and both parents alive), the quality of the caregiver-child relationship (with higher scores denoting higher levels of conflict between the caregiver and the child), who the child lives with, and whether there is sufficient food in the household. These variables were added to the stage two model, and variables that were not significant at the 15% level were removed using a backward elimination algorithm.

At the fourth stage, child illness factors were considered for inclusion. The candidate variables were whether or not the child was born prematurely, the child's current “cluster of differentiation 4” (CD4) count [22], the child/adolescent's body mass index (BMI) [22], the highest WHO stage reached by the child, whether or not the child/adolescent was on ART, and the amount of pain experienced by the child/adolescent (as measured by the Wong-Baker face scale). The child illness variables were added to the stage 3 model, and those that were not significant at the 15% level were removed using a backward elimination algorithm.

At the fifth and final stage, psychiatric comorbidities (including behavioral disorders) and the other mood disorders, e.g., any depression (in the case of the model for any anxiety), were considered for inclusion. These variables were added to the model, and those that were not significant at the 10% level were removed using a backward elimination algorithm.

The impact of mood disorders on negative clinical or behavioral outcomes was investigated by fitting logistic regression models or ordinal logistic regression models. Logistic regression models were fitted for sexual debut, poor academic performance, disciplinary problems at school, any hospital admission, and having missed at least one dose of ART in the previous three days. Ordinal logistic regression models were fitted for the number of school days missed in the past month, for the number of visits to a health facility in the past month, and for the number of doses of cotrimoxazole prophylaxis missed in the past month). The models were adjusted for study site, age of the child/adolescent, sex of the child/adolescent, and education and included the four-level behavioral disorder group.

Prevalence of the two disorders was determined as the number of people in the sample with the characteristic of interest (positive for the disorder), divided by the total number of people in the sample.

There was no adjustment for multiple testing, so the findings should be interpreted with caution as exploratory findings [20]. All analyses were carried out using Stata release 15.

### 2.5. Ethical Considerations

The study obtained ethical approvals from the Uganda Virus Research Institute's Research and Ethics Committee (Ref: GC/127/14/04/459), the Ethics Committee of the London School of Hygiene and Tropical Medicine, and the Uganda National Council of Science and Technology (Research Registration number: HS 1601). Participants found to have a psychiatric disorder were provided with psychoeducation and referred to their local mental health departments.

## 3. Results

### 3.1. Characteristics of Study Participants

Of the 1,339 CA-HIV enrolled into this study, 64% were aged 5 and 11 years, and 36% were 12-17 years (Table 2). The urban and rural study sites contributed 51% and 49% of participants, respectively. Males and females enrolled in the study were 48% and 52%, respectively. Seventy-nine percent (79%) of the respondents were Christians, 20% Muslims, and 1% reported other religions. More than three quarters reported to had attained at least primary level education, 79% had CD4 counts equal or greater than 500 cells/*μ*L, and 95% were receiving ART.

### 3.2. Prevalence of Anxiety Disorders

In total, 120 CA-HIV (9.0%, 95% CI) had an anxiety disorder by symptom count. Of these 36 CA-HIV (2.7%, 95% CI) had a clinical anxiety disorder (Table 3).

### 3.3. Prevalence of Depressive Disorders

In total, 85 CA-HIV (6.4%, 95% CI95%CI) had a depressive disorder by symptom count, and 39 CA-HIV (2.9 %, 95% CI95%CI) had a clinical depressive disorder (Table 3).

### 3.4. Clinical Correlates for Anxiety Disorders

Any anxiety disorders were associated with caregiver distress indicated by the SRQ-20 score (aOR 1.12, 95% CI 1.07–1.15; *p* < 0.001), current CD4 count (aOR 1.00, 95% CI 1.02–1.05; *p* = 0.021), age of the child (CA-HIV 12-17 years were protective by 38% against anxiety disorders; aOR 0.62, 95% CI 0.48–0.88; *p* = 0.003), caregiver religion (*p* = 0.015), attention-deficit/hyperactivity disorder (aOR 3.31, 95% CI 2.03-4.78; *p* < 0.001), oppositional defiant disorder (aOR 4.34, 95% CI 2.64–7.10; *p* < 0.001), depressive symptoms (aOR 3.06, 95% CI 1.78 - 4.21; *p =* 0.001), and having experienced “extremely upsetting events” (aOR 2.24,95% CI 1.61–3.26; *p* < 0.001) (Table 4).

### 3.5. Clinical Correlates of Depression

Depressive disorders were associated with the study site (living in rural area was protective by 61% against depressive symptoms; aOR 0.39, 95% CI 0.24–0.56; *p* < 0.001), age of the child (CA-HIV in the age bracket of 12-17 years had a five-fold increased risk of having depressive symptoms; aOR 4.46, 95% CI 2.93–6.77; *p* < 0.001), caregiver age (*p* = 0.028), caregiver distress indicated by SRQ-20 (the higher the caregiver distress, the more likely the CA-HIV was to have a depressive disorder; aOR 1.13, 95% CI 1.06-1.17; *p* < 0.001), quality of the child-caregiver relationship (aOR 1.50, 95% CI 1.09-2.02; *p* = 0.012), and co-occurring anxiety disorders (aOR 1.87, CI 1.18-2.90; *p* = 0.007). The odds of a depressive disorder were lower for a CA-HIV with a caregiver aged ≥ 50 years (*p* = 0.028) (Table 4).

### 3.6. Effect of Anxiety and Depression on Clinical Outcomes

There was no evidence that anxiety and depressive disorders were associated with poor academic performance, disciplinary problems at school, school days missed in the past month, and visit to the health unit (see Table 5). CA-HIV with an anxiety disorder were more likely to have had at least a hospital admission (aOR = 2.58; 95% CI 1.16–5.42; *p* = 0.02); similarly, CA-HIV with a depressive disorder were more likely to have had at least a hospital admission (aOR = 2.47; 95% CI 1.93–6.52; *p* = 0.041) Table 5.

## 4. Discussion

This study aimed to determine the prevalence of anxiety and depressive disorders among CA-HIV in Uganda and their associations with functioning. The prevalence of anxiety disorders was 9% while that of depression was 6.4%. The correlates of anxiety and depressive disorders included age of the child, caregiver' psychological distress, caregivers' age, child-caregiver relationship, child's current CD4 count, and hospital admissions. Anxiety and depressive disorders were associated with frequent hospital admissions for CA-HIV.

### 4.1. Prevalence of Anxiety Disorders

According to this study, the prevalence of anxiety problems (based upon the symptom count criteria) was 9.0%—similar rate previously reported by the same study group [23], whereas a prevalence of 32.2% was reported by the authors in Kenya [14]; the difference between the prevalence rates reported by the two studies (Ugandan CHAKA study and urban Kenyan study among CA-HIV) possibly results from the use of different instruments; the Ugandan study used the Child and Adolescent Symptom Inventory-5 (CASI-5) [16], whereas the Kenyan study used the Mini international Neuropsychiatric Interview for children and adolescents (MINI Kid) [14]. Based upon the symptom and impairment criteria for DSM-5 (using the CASI-5 used in the CHAKA study and a similar psychiatric disorder instrument (CASI-4R) used in other studies conducted among CA-HIV), the prevalence of anxiety (2.7%) established by this study was similar to the reported prevalence in the USA of 3% [2] for anxiety disorders (based upon the symptom and impairment criteria). Similarly, other studies in the USA have established varying rates of anxiety disorders among CA-HIV: 46% [24], 3% [2], and 1% [1]. Probably, differences between the prevalence rates of anxiety disorders established by several studies result from the use of different study instruments; Mellins et al. [24] used the caregiver and youth versions of the Diagnostic Interview Schedule for Children (DISC-IV) to study perinatally HIV-infected (HIV+) adolescents (aged 9 to 16 years), whereas Gadow et al. [2] and Nachman [1] used the Child and Adolescent Symptom Inventory-4R (CASI-4R) [25] to study youths perinatally infected with HIV with a comparison sample of peers exposed to HIV. Another major difference responsible for the varying prevalence rates of anxiety disorders among CA-HIV in USA and other parts of the world is the population studied; the USA HIV population is much different from the Uganda population. Relatedly, variation in the prevalence rates of anxiety disorders among CA-HIV in USA and other parts of the world can be explained by the mode of transmission; in USA parental transmission, IV drug use is much more common.

### 4.2. Prevalence of Depressive Disorders

Based upon the symptom count criteria, about 6.4% of the participants in this study had significant depressive symptoms, including major depressive disorder (MDD) with a rate of 3.9%. A rate of 2% for MDD was reported in western Kenya [26], 2% reported by one study in the USA [1], although a much higher rate of 17.8% was reported in urban Kenya [14]. Similarly, other studies among children and adolescents conducted in the sub-Saharan region established higher prevalence rates of depression, 18.9% in Malawi [27, 28] and 25% in Rwanda [29], but all these studies used different instruments [30] from the CASI-5 [16] which was used during the CHAKA study. Based upon the symptom and impairment criteria for DSM-5 (using the CASI-5 used in the CHAKA study and a similar psychiatric disorder instrument (CASI-4R) used in other studies conducted among CA-HIV), the prevalence of depression (2.9%) established by this study was similar to the reported prevalence in the USA of 2% [1] for major depressive disorders (based upon the symptom and impairment criteria). Unsurprisingly, adolescents living with HIV face particular emotional, behavioral, and mental health challenges, compared to those without HIV [30]. Thus, the etiology of depression among children/adolescents with HIV extends beyond their HIV infection and is possibly driven by the full range of their biopsychosocial experiences [30].

The high prevalence of anxiety and depressive disorders established by this study among CA-HIV compared to HIV-uninfected children/adolescents (in East Africa) could be a result of stigma, children/adolescents transitioning their medical care to adult care settings, and disclosure of their HIV status, coupled with the increased burden and interplay of physical, emotional, and social stressors during this vulnerable, developmental period [30, 31]. Similarly, the high prevalence rates of anxiety and depression established by this study probably relates to the negative life events, coupled with other causal factors that result in predisposition to anxiety and depressive disorders [14, 32]. In this study, CA-HIV had experienced substantial family loss, with about half of them being orphaned and only a quarter living with biological parents [23]. The etiology of anxiety and depressive disorders relates to the complex interactions of genetic, social, and environmental factors [32].

### 4.3. Clinical Correlates for Anxiety Disorders

Sociodemographic and clinical factors significantly associated with anxiety disorders in this study were as follows: age of child, caregiver distress (indicated by the SRQ-20 score), current CD4 count of the CA-HIV, and caregiver religion. This study established that the age of the child was significantly associated with anxiety disorders among CA-HIV. Findings from this study happen to be in agreement with other previous studies [30, 33], which suggest that CA-HIV are at an increased risk of having anxiety-related problems as compared to non-HIV-positive children and adolescents but with advancing age of CA-HIV, the “anxiety-related problems” are lessened through regular contact with health services and mental health support [34]. According to this study, caregiver psychological distress was significantly associated with anxiety disorders among CA-HIV; a previous publication by the same research group established that increased caregiver psychological distress is associated with increased emotional (anxiety) related problems among CA-HIV due to the multiple challenges experienced by both the caregivers and CA-HIV [33]. According to this study, current CD4 count of CA-HIV was associated with anxiety disorders, consistent with other previous studies which suggest that low CD4 count is associated with major depression and anxiety among CA-HIV [1, 31, 35]; anxiety among CA-HIV relates to the “fear and anxiety” associated with the likely clinical manifestation of HIV/AIDS [30]. This study revealed an association between caregiver religion and anxiety disorders among CA-HIV; findings from this study relates to a previous study which suggested that in Africa, religion and life are so deeply fused to the extent that material utility of religion in search for solutions to life's crises happens to be an everyday experience to most caregivers to CA-HIV [36]; thus, they often use it as a coping strategy [33]. In this study, anxiety disorders were associated with any attention-deficit/hyperactivity disorder (ADHD), oppositional defiant disorder(ODD), any depression, and extremely upsetting events. Studies undertaken in both Kenya [14] and the USA [7, 24] have reported rates of psychiatric comorbidity among CA-HIV of between 26% and 44%. Likewise, studies conducted elsewhere have established that anxiety and depressive disorders are comorbid with other behavioral problems among CA-HIV [35, 37, 38].

### 4.4. Clinical Correlates of Depression

Similarly, sociodemographic and clinical factors significantly associated with depressive disorders (based upon the DSM-5 symptom count criteria) in this study were as follows: study site, age of the child, caregiver age, caregiver distress (indicated by the SRQ-20 score), quality of the child-caregiver relationship, and with the CA-HIV having an anxiety disorder. According to the results from this study, living in a rural setting was protective against depressive symptoms among CA-HIV; findings from this study are in agreement with a previous study which established that rural settings may be protective against the development of mental disorders [39], but findings are contrary to a study which established that residing in a rural setting is significantly associated with depressive symptoms among CA-HIV [40]; a related study suggested that depression is significant in the rural population among people in the lower socioeconomic class [41, 42]. A plausible explanation for the findings from this study (living in a rural setting being protective against depressive symptoms among CA-HIV) relates to the potential buffering by social support systems [36, 43], since these CA-HIV were receiving an instrumental support from other relatives living within their rural setting; thus, the material support from relatives buffered these CA-HIV against hardships that could subject them to depressive symptoms [23]. Relatedly, this study established that adolescents had a five-fold increased risk of having depression; findings are in agreement with previous studies which established that increasing age among children and adolescents with HIV/AIDS is associated with depression, largely due to biological, social, and psychological dynamics [28, 40, 44]. An extensive review of studies among PHIV+ youth aged 10 years and older established that age was associated with the presence of “anxiety and depressive disorders” [31]. This study further revealed that the odds of a depressive disorder were lower for a CA-HIV with a caregiver aged over fifty years; a related study established that increasing caregiver age is associated with decreased ratings of emotional problems among CA-HIV [33], which happens to be a result of the experience and emotional maturity of caregivers with advanced age [36], thus, the lower odds of depressive disorders among CA-HIV with a caregiver aged over fifty years of age. Similarly, findings from this study suggest that caregiver distress was significantly associated with depressive disorders among CA-HIV; possibly, caregivers' psychological distress (indicated by the SRQ-20 score) compromises normal parenting, negatively impacts on the emotional development of CA-HIV, and thus makes them to be at risk of developing disorders like depression [6]. Similarly, a study among non-HIV children/adolescents established that caregivers' mental health (psychological distress) is associated with relatively high levels of anxiety and depression since witnessing poor health and incapacitation in caregivers is stressful for children [45]. This study established that quality of the child-caregiver relationship was significantly associated with depressive disorders among CA-HIV; a previous study suggested that caregiver-child stress (including factors such as parent-child relationship problems, caregiver mental health problems, and stressful or negative life events) happens to be associated with both emotional problems (including depression) among CA-HIV [33]; relatedly, other previous studies revealed that child-caregiver interaction is associated with depression among CA-HIV [6, 11, 31]. In this study, depressive disorders were associated with any anxiety, which relates to findings established by other studies among CA-HIV [7, 14, 24].

Sociodemographic and clinical factors significantly associated with anxiety and depressive disorders relate to the abundance of risks and potential pathways to poor mental health among CA-HIV, coupled with a constellation of biomedical, genetic, familial, and environmental characteristics [31].

### 4.5. Effect of Anxiety and Depression on Clinical Outcomes

In this study, there was no evidence that anxiety and depressive disorders were associated with poor academic performance, disciplinary problems at school, school days missed in the past month, and visit to the health unit in the past month. In this study, CA-HIV with an anxiety disorder was more likely to have been admitted to hospital. Possible explanation could be that CA-HIV constantly think about potential mortality, which subjects them to the anxiety disorders, thus the health-seeking behavior and hospitalization [13].

### 4.6. Strength and Limitations

This study has several strengths to include an exceptionally large sample of CA-HIV and use of a comprehensive assessment battery, but there are also limitations. Because our analyses are cross-sectional, we cannot comment on the causal directions, but these will be addressed in future publications about the longitudinal component of the CHAKA study. Since anxiety and depressive symptoms are influenced by environmental variables and therefore different informants can disagree about symptom severity [14, 46], our prevalence rates for anxiety and depressive disorders in Uganda should be considered conservative estimates since we did not obtain the CASI-5 ratings from school teachers. The exclusion of the child and adolescent self-report data is a limitation of this study; it would have been prudent to consider defined anxiety and depressive disorders that additionally met the symptom count cutoff according to the child/adolescent self-report. Therefore, the results are entirely based upon caregiver' reports. Because we do not have a comparable sample of sero-negative children/adolescents from the same geographic areas and environment, it is not possible to know whether relations between anxiety, depressive disorders, and clinical outcomes are influenced by HIV status. This does not, however, lessen our findings for CA-HIV. By design, the present study focused upon CA-HIV living in Uganda, and owing to considerable cultural variation in East Africa, our results may not generalize to other countries in the region.

## 5. Conclusions

About a seventh of the children and adolescents with HIV in this study presented with significant anxiety and depressive disorders. Previous work by this research group and others has shown that subthreshold psychiatric disorder are not only a significant predictor of future psychiatric disorder [47, 48], but have been associated with increased disability and many other negative consequences [49]. In this study, anxiety and depressive disorders spanned a broad spectrum of psychopathology. Apart from age of the child, caregivers' distress (indicated by the SRQ-20 score), caregivers' age, child-caregiver relationship, and current CD4 count, all other investigated sociodemographic and clinical factors were not significantly associated with anxiety and depressive disorders. Lastly, anxiety and depressive-related problems were associated with significant psychiatric comorbidity.

### 5.1. Clinical, Policy, and Research Implications

This study has several strengths to include an exceptionally large sample of children and adolescents infected with HIV/AIDS (CA-HIV) [50]; to the best of our knowledge, this is the largest study to date on emotional and behavioral disorders among CA-HIV in Uganda [23]. The implications of these findings are many. Firstly, given the considerable burden of anxiety, depressive disorders, and related problems (including psychiatric disorders) among CA-HIV in this study, there is an urgent need to integrate mental health services into routine HIV care of CA-HIV in sub-Saharan Africa, in line with WHO recommendations [51]. Secondly, since CA-HIV suffered from psychiatric morbidity spanning a broad spectrum, any mental health intervention program that is to be implemented in HIV care services for CA-HIV should cater for a broad spectrum of psychopathology. Thirdly, since most of the investigated sociodemographic and clinical factors were significantly associated with anxiety and depressive disorders, it is possible to build a profile for those at risk for anxiety and depressive-related problems among CA-HIV. Therefore, interventions for anxiety and depressive-related problems among CA-HIV should be based upon case detection through the deployment of screening tools that are easy to use. Fourthly, due to the high rates of psychiatric comorbidity, mental health screening and treatment programs should be comprehensive to ensure assessment of the entire spectrum of anxiety and depressive-related problems. Lastly, there is a need to develop and evaluate interventional models that will integrate mental health into HIV care services for CA-HIV in sub-Saharan Africa. However, these models should take into consideration the above study findings and the unique challenges facing health systems in sub-Saharan Africa which include the following: lack of demand for formal mental health services, a severe shortage of mental health professionals, a reluctance of primary care providers to engage in mental health care, and a severe shortage of primary care workers in most public health units [52, 53].

## Figures and Tables

**Table 1:** Data collection tools for the study.

	Instrument used	Description	Questions or categories (examples)	Remarks	Reference
Sociodemographic variables					
	Structured sociodemographic questionnaire; study site, age, gender, ethnicity, educational level attained, socioeconomic status (SEI), and religion	Socioeconomic index (SEI) was constructed from commonly available household items in typical Ugandan households and has previously been used by this (Kinyanda et al., 2011c)	To assess SEI, e.g., *does your household have electricity?* Response: Yes/no	Administered to caregiver Has previously been used by this study group	Kinyanda et al., 2011a; 2012a

Caregiver variables					
	Caregivers' sociodemographics; age, gender, highest level of education, marital status, and caregiver HIV status	Questions that gathered information about caregivers' sociodemographics	To assess for caregivers' highest level of education, e.g., *What is the highest level of education attained by caregiver?* Response: 1 = no formal education 2 = primary level 3 = secondary level 4 = university level 5 = other tertiary level	Administered to caregiver Has previously been used by this study group	Kinyanda et al., 2011a; 2012a
To assess caregivers' psychological distress	WHO Self-Report Questionnaire; WHO SRQ-20	20-item questionnaire that assesses general psychological distress	Has items such as: *Do you often have headaches?* Response: Yes/no	Administered to caregiver A total score on the SRQ 20 is then calculated for each patient, with a clinically significant cutoff of ≥6	(WHO SRQ 20; WHO, 1994) Was culturally validated and used in Uganda by Nakimuli-Mpungu et al., 2012

Childhood's psychosocial environment factors					
Felt HIV stigma	Brief HIV stigma scale (B-HSS)	9-item questions on experiences, feelings, and opinions on HIV stigma; five items were used	Has items such: *I have been discriminated against at school/work because of being HIV positive* Response: Yes/no	Administered to adolescents This 9-item scale is a psychometrically valid and reliable instrument that has both clinical and research applications	Berger et al., 2001 Mavie, 2014
Child-caregiver relationship	The caregiver-child interaction scale (CCIS) was used to assess for the child-caregiver relationship	The caregiver-child interaction is a 10-item, self-administered, communication scale. It was adapted from the child's report of parental behavior inventory	The items were scored as follows; 1 = never, 2 = rarely, 3 = sometimes, 4 = often, and 5 = always	In this study, four questions were used This was administered to caregivers	Margolis and Weintraub, 1977
Trauma	The childhood trauma questionnaire-short form (CTQ-SF)	28-item questionnaire on traumatic events	Selected examples: *Have you ever been beaten, hit, and slapped?* Response: 1 = yes 2 = no	Two items were used in this study This was administered to adolescents Employed for the second time in Uganda by Kinyanda et al., 2016	Bernstein and Fink, 1998
Food security	One-item question	Closed question	The item was: *In the last month, did you or your family have enough food?* Response: Yes/no	This was administered to caregivers It was previously used in the HIV situation of Uganda by Kinyanda et al., 2011	Kinyanda et al.,2011a;2012a
Negative life Events	The modified European Parasuicide Interview Schedule	27-item questions on adverse life events experienced in the last one year with related to parents, sibling, children, and self; three items were used in this study	*The two questions asked were:* (i) Did your parents separate? (ii) In the last year, was either of your parents seriously ill? e.g., got admitted *Responses:* *Yes/no*	This was administered to adolescents Two questions on adverse life events experienced in the last one year This tool has previously adapted to the Ugandan sociocultural context and used in HIV research	Kerkhof et al., 1989; Kinyanda et al., 2005

Child illness variables					
WHO clinical stage for HIV/AIDS	WHO clinical staging criteria		Respondents classified as Stages I to IV based on the presence/absence a combination of 17 HIV associated clinical symptoms	This was administered to adolescents	[22]
CD4 counts	CD4 count taken in the last 6 months		Cells/*μ*l of blood		
Viral load	Viral load determined at assessment		Copies/ml		

Dependent variables					
Anxiety and depressive disorders	DSM-5-referenced, behavior rating scale and the Child and Adolescent Symptom Inventory-5 (CASI-5)	Anxiety and depressive disorders presentations considered, CA-HIV was regarded as having anxiety disorder and depression if he/she reached the cutoff for both anxiety disorders and depression, while a CA-HIV was regarded as having an anxiety disorder if they reached the cutoff for anxiety disorders; CA-HIV was regarded as having depression if they reached the cutoff for depression	CA-HIV was considered to have the disorder if the number of symptoms in the category for the disorder (anxiety or depression) which the caregiver rated as occurring “often” or “very often” reached a predetermined cutoff Selected example: *Is extremely tense or unable to relax* Responses; 0 = never 1 = sometimes 2 = often 3 = very often	Six items of the category D of the CASI-5 were used to assess for “anxiety disorder” presentations	[15]

Negative clinical and behavioral outcomes					
Academic performance	This section assessed the academic performance of the CA-HIV at school	Since academic performance in the Uganda education system is measured differently at the primary and secondary levels, we used 3 questions to develop a composite measure of poor academic performance. The 3 questions were: *What was the academic performance of this child last term/semester?* Response: 1 = poor 2 = fair 3 = good 4 = excellent *What academic position did this child/adolescent hold in class last term/semester?* Response: Out of how many pupils/students ………………. Number *What aggregate points did this child/adolescent attain last term/semester?* Aggregate points attained………….	Poor academic performance at school which was determined as follows: In certain classes, performance is measured by a “points” aggregate, with lower aggregates denoting better performance. If the ratio of the points obtained to the best possible aggregate was greater than 12, then the CA-HIV was deemed to have performed poorly Alternatively, if a point aggregate was not available, the CA-HIV was deemed to have performed poorly if his or her position in class was in the fourth quartile. If neither of these was available, then the performance in class was determined by the answer “poor” to the question “what was the academic performance of this child last term/semester?”	Asked of the caregiver Was adapted and used in Uganda by Kinyanda et al.2014	[1]
Experienced problems at school	This section assessed for social functioning of the CA-HIV at school	Used 3 questions to develop the composite measure of “having experienced problems at school.” The 3 questions were: *Did the child/adolescent suffer disciplinary measures (including suspension, and dismissal) in the last term/semester?* Response: Yes/no *Did the child/adolescent stay away from school without permission in the last term/semester?* Response: Yes/no *Number of days missed at school in the last term* Response: Number ………	A CA-HIV was deemed to be positive for the composite measure, “having experienced problems at school” if any of the following three conditions were met: (i) A positive answer to the question “did the CA-HIV suffer disciplinary measures (including suspension/dismissal) in the last term/semester?” (ii) A positive answer to the question “did the CA-HIV stay away from school without permission in the last term/semester?” (iii) The pupil was absent from school for 6 or more days in the last term/semester	Asked of the caregiver Was adapted and used in Uganda by Kinyanda et al.2014	[1]
Risky sexual behavior	Involvement in sexual activity	Assessed sexual debut	An example of items; *Have you ever had sex?* *Response:* *Yes/no*	This was administered to adolescents This has previously used by Kinyanda et al., 2011	Employed For the second time in Uganda by Kinyanda et al., 2016
Frequency of visits to the health unit	One item was used	Number of times visited to the health unit in the past month	Item was as follows: *How many times did you visit the health unit in the last month?* *Response:* *Number of visits……………*	This administered to adolescents only	Employed for the second time in Uganda by Kinyanda et al., 2016
Frequency of hospital admissions	One item was used	Used question: *For how many days were you admitted to hospital in the last 6* months*?* Response: Number of days…………. To create a derived variable.	Used responses to the question: *For how many days were you admitted to hospital in the last 6 months?* To create a derived variable of whether or not the CA-HIV has been admitted to hospital in the last month	This administered to adolescents	Employed for the second time in Uganda by Kinyanda et al., 2016
Missed prophylaxis or ART (poor adherence to HIV treatment)	Used the 3-day recall test to assess for non-adherence to HIV treatment	Used two questions to arrive at the composite measure of “being non-adherent to HIV treatment'” The two questions were as follows: *For those on ART: How many days in the past 3 days have you missed taking ART?* Response: ………. Number of days *For those on Septrin/Dapsone: How many days in the past 3 days have you missed taking Septrin/Dapsone?* Response: ………. Number of days….	A CA-HIV had to meet the following conditions to be assessed as non-adherent to HIV treatment. If the participant was on ART, then failure to adhere was defined as having missed a dose of ART in previous three days. If the participant was not yet on ART, then failure to adhere was defined as having missed a dose of cotrimoxazole (CTX) prophylaxis in the previous three days	This was administered to adolescents only	Employed for the second time in Uganda by Kinyanda et al., 2016

**Table 2:** Characteristics of study participants.

Variable	Level	Total (*n* = 1,339) *n* (%)	Children (*n* = 855) *n* (%)	Adolescents (*n* = 484) *n* (%)
Study site	Urban	684 (51.1%)	419 (49.0%)	265 (54.7%)
	Rural	655 (48.9%)	436 (51.0%)	219 (45.2%)

Sex	Male	638 (47.7%)	412 (48.2%)	226 (46.8%)
	Female	699 (52.3%)	442 (51.8%)	257 (53.2%)

Religion	Christian	1058 (79.0%)	672 (78.6%)	386 (79.7%)
	Muslim	273 (20.4%)	178 (20.8%)	95 (19.6%)
	Others/missing	8 (0.6%)	5 (0.6%)	3 (0.6%)

Tribe	Baganda	967 (72.3%)	625 (73.2%)	342 (70.8%)
	Non-Baganda	370 (27.7%)	229 (26.8%)	141 (29.2%)

Child lives with	Both parent	354 (26.4%)	255 (29.8%)	99 (20.4%)
	Single parent	512 (38.2%)	337 (39.4%)	175 (36.2%)
	Grandparents	258 (19.3%)	167 (19.5%)	91 (18.8%)
	Others/missing	215 (16.1%)	96 (11.2%)	119 (24.6%)

Orphan hood	Single parent orphan	446 (34.5%)	247 (30.0%)	199 (42.2%)
	Double parent orphan	127 (9.8%)	40 (4.9%)	87 (18.4%)
	Non-orphan	721 (55.7%)	535 (65.1%)	186 (39.4%)

Highest level of education attained	No formal	29 (2.2%)	12 (1.4%)	17 (3.5%)
	Preprimary	217 (16.2%)	214 (25.2%)	3 (0.6%)
	Primary	954 (71.5%)	624 (73.2%)	330 (68.3%)
	Secondary	132 (9.9%)	1 (0.1%)	131 (27.1%)
	Missing	3 (0.2%)	1 (0.1%)	2 (0.4%)

Socioeconomic index	Mean(Std)	4.4 (1.8)	4.2 (1.8)	4.8 (1.8)

Socioeconomic index (grouped)	0–2	194 (14.5%)	146 (17.1%)	48 (9.9%)
	3–4	480 (35.8%)	328 (38.4%)	152 (31.4%)
	5–6	480 (35.8%)	281 (32.9%)	199 (41.1%)
	7–9	185 (13.8%)	100 (11.7%)	85 (17.6%)

Baseline CD4 counts (cells/*μ*l)	<500	265 (19.8%)	106 (12.4%)	159 (32.8%)
	≥ 500	1060 (79.2%)	742 (86.8%)	318 (65.7%)
	Missing	14 (1.0%)	7 (0.8%)	1 (1.5%)

Child on ART at baseline?	Yes	1277 (95.4%)	818 (95.7%)	459 (94.8%)
	No	62 (4.6%)	37 (4.3%)	25 (5.2%)

**Table 3:** Anxiety and depressive disorders by child category.

Psychiatric disorder∗	Total (*N* = 1339) *n*(%) (95% CI)		Children (*n* = 860) *n*(%) (95% CI)	Adolescents (*n* = 479) *n*(%) (95% CI)
	Psychiatric disorders€ (based on both symptom and functional impairment criteria)	Psychiatric problems∞	Psychiatric problems∞	Psychiatric problems∞
Any anxiety, depressive disorder∗	53(4.0%) (2.8–5.2)	183(13.7%) (11.9–15.6)	69(8.1%) (6.4–10.1)	114(23.5%) (19.9–27.5)
Any anxiety disorder∗	36(2.7%) (1.5–3.2)	120(9.0%) (7.5–10.6)	49(5.7%) (4.3–5.7)	71(14.7%) (11.8–18.1)
Generalized anxiety disorder	25(1.9%) (1.1–2.3)	52(3.9%) (3.0–5.1)	17(1.9%) (1.2–3.2)	35(7.2%) (5.2–9.9)
Specific phobia∗	—	257(19.2%) (17.2–21.4)	144(16.8%) (14.5%-19.5%)	113(23.3%) (19.8%-27.3%)
Panic disorder∗	—	74(5.5%) (4.4%-6.9%)	21(2.4%) (1.6%-3.7%)	53(10.9%) (8.4% - 14.1%)
Social anxiety disorder	37(2.8%) (2.4–3.2)	44(3.3%) (2.4–4.4)	10(1.2%) (0.6–2.1)	34(7.0%) (5.1–9.7)
Separation anxiety disorder	20(1.5%) (1.3–1.9)	54(4.0%) (3.1–5.2)	28(3.3%) (2.3–4.7)	26(5.4%) (3.7–7.8)
Any depressive disorder	39(2.9%) (2.3–3.4)	85(6.4%) (5.1–7.8)	30(3.5%) (2.5–5.0)	55(11.4%) (8.8–14.5)
Major depressive disorder	14(1.0%) (0.7–1.3)	52(3.9%) (3.0–5.1)	27(3.2%) (2.2–4.6)	25(5.2%) (3.5–7.5)
Persistent depressive disorder	13(0.9%) (0.6–1.2)	36(2.7%) (1.9–3.7)	5(0.6%) (0.2–1.4)	31(6.4) (4.5–8.9)

Note: ∗The DSM-5 column does not have entries for the disorders: panic disorder and specific phobia which in the CASI-5 do not have functional impairment scales. ^€^Psychiatric disorders (met symptom and impairment criteria for DSM-5). ^∞^Emotional and behavioral problems (met only symptom criteria for DSM-5).

**Table 4:** Factors associated with anxiety and depressive disorders: results of fitting logistic regression models including comorbidities.

Outcome	Parameter	Any anxiety aORs; 95% CI; *p* value	Any depression aORs; 95% CI; *p* value
Site	Urban	1	1
	Rural	0.82 (0.0.62–1.02)	0.39(0.24–0.56)
		*p* = 0.185	*p* < 0.001

Sex of child	Male	1	1
	Female	1.06 (0.80–1.20)	1.35 (0.89–2.00)
		*p* = 0.483	*p* = 0.148

Age of child	5–11 years	1	1
	12–17 years	0. 62 (0.48–0.88)	4.46 (2.93–6.77)
		*p* = 0.003	*p* < 0.001

SES score	Per unit increase	1.04 (0.96–1.10)	—
		*p* = 0.118	

Education level of child	No formal	1	—
	Preprimary	1.60 (0.48–5.98)	
	Primary	2.30 (0.64–7.31)	
	Secondary	1.68 (0.48–6.58)	
		*p* = 0.315	

Caregiver religion	Christian	1	1
	Muslim	0.71 (0.51–0.98)	1.21(0.65–2.24)
	Other/missing	0.23 (0.05–0.92)	1
		*p* = 0.015	*p* = 0.432

Caregiver SRQ-20 score	Per unit increase	1.12(1.07–1.15)	1.13(1.06–1.17)
		*p* < 0.001	*p* < 0.001

Child WHO stage	I	1	—
	II	0.99 (0.67–1.46)	
	III	0.72 (0.47–1.12)	
	IV	1.29 (0.53–3.14)	
		*p* = 0.234	

Current CD4 count	Per 100 cell increase	1.00 (1.02–1.05)	—
		*p* = 0.021	

Quality of caregiver relationship	Per unit increase	—	1.50(1.09–2.02)
			*p* = 0.012

Caregiver age (grouped)	≤24	—	1
	25–34		2.19(0.80–5.97)
	35–49		1.34(0.49–3.40)
	≥ 50		0.65(0.21–2.18)
			*p* = 0.028

Caregiver occupation	Farmer/fish	—	—
	Professional		
	Other		

Who does child live with	Both parents	—	—
	Mother		
	Father		
	Others		

Body mass index	Both parents	—	—
	Mother		
	Father		
	Others		

Any anxiety	Yes	—	1.87(1.18–2.90)
			*p* = 0.007

Any ADHD	Yes	3.31 (2.03–4.78)	—
		*p* < 0.001	

ODD	Yes	4.34 (2.64–7.10)	—
		*p* < 0.001	

Any depression	Yes	3.06 (1.78–4.21)	—
		*p* < 0.001	

Extremely upsetting events	Yes	2.24 (1.61–3.26)	—
		*p* < 0.001	

**Table 5:** Outcomes associated with “anxiety and depressive disorders”.

Outcome	Parameter	Any anxiety aOR; 95% CI; *p* value	Any depression aOR; 95% CI; *p* value
Sexual debut∗	Binary	0.45 (0.09-1.72)	1.67 (0.65–4.69)
	Ever had sex	*p* = 0.2	*p* = 0.264

Poor academic performance	Binary	2.62 (0.85–8.04)	1.15 (0.38–3.46)
	Yes(poor)	*p* = 0.09	*p* = 0.81

Disciplinary school problems	Binary	0.94 (0.41–2.19)	1.66 (0.60–4.55)
	Yes (problems)	*p* = 0.89	*p* = 0.32

School days missed in past month	Ordinal	1.02 (0.98–1.07)	1.04 (0.98–1.11)
	Missed more	*p* = 0.23	*p* = 0.15

Visit to the health unit	Binary	0.90 (0.57–1.44)	0.93 (0.48–1.79)
	Yes	*p* = 0.67	*p* = 0.82

Any hospital admission	Binary	2.58 (1.16–5.42)	2.47 (1.93–6.52)
	Yes	*p* = 0.02	*p* = 0.041

Missed Cotrimoxazole and prophylaxis∗	Ordinal	1.07 (0.77–1.49)	1.03 (0.71–1.49)
	Missed more doses	*p* = 0.66	*p* = 0.87

Missed any ART dose∗	Binary	1.10 (0.46–2.62)	1.09 (0.42–2.74)
	Yes (missed)	*p* = 0.83	*p* = 0.86

∗Assessed only among adolescents.

## Data Availability

All data and materials in this manuscript, additional files, and figures attached are freely available with no restrictions.

## Additional Points

*Submission Declaration.* The work described has not been published previously, it is not under consideration for publication elsewhere, and its submission for publication by Journal of Affective Disorders is approved by all authors.
